# Similarity Estimation Between DNA Sequences Based on Local Pattern Histograms of Binary Images

**DOI:** 10.1016/j.gpb.2015.09.007

**Published:** 2016-04-27

**Authors:** Yusei Kobori, Satoshi Mizuta

**Affiliations:** Graduate School of Science and Technology, Hirosaki University, Hirosaki, Aomori 036-8561, Japan

**Keywords:** Genome sequence, Mitochondria, Bitmap image, Occurrence frequency, Distance measure

## Abstract

Graphical representation of DNA sequences is one of the most popular techniques for alignment-free sequence comparison. Here, we propose a new method for the feature extraction of DNA sequences represented by binary images, by estimating the similarity between DNA sequences using the frequency histograms of local bitmap patterns of images. Our method shows linear time complexity for the length of DNA sequences, which is practical even when long sequences, such as whole **genome sequences**, are compared. We tested five **distance measures** for the estimation of sequence similarities, and found that the histogram intersection and Manhattan distance are the most appropriate ones for phylogenetic analyses.

## Introduction

Sequence alignment [1], [2] is generally used to estimate similarities between relatively short sequences less than about several thousand characters, such as nucleotide sequences or amino acid sequences. However, the time complexity of the alignment is the square of the sequence length, thus the long sequence length may result in enormous amount of computation time [3]. Therefore, to reduce the time required for comparing long sequences such as whole genome sequences, developing so-called alignment-free methods becomes a necessity.

Graphical representation of biological sequences represents one of the most popular methods for the alignment-free sequence comparison [4]. Various methods based on graphical representation have been introduced, and almost all methods share the common basic procedure. Every base type in a DNA sequence is replaced by an individual vector in a two-dimensional (2D) [5], [6], three-dimensional (3D) [7], [8], or even higher-dimensional [9] expression space. These vectors are then connected successively, drawing a trajectory in the expression space and finally, the distances between the trajectories, or graphs, are calculated according to a predefined distance measure. While there exist many methods in the field of graphical representation of biological sequences as mentioned above, further improvement in terms of the performance and the calculation time is still required.

In this study, we propose a new method for sequence comparison based on the graphical representation. We expressed a DNA sequence as a binary image—each pixel of a binary image was plotted in either black or white—in a two-dimensional space, and counted the occurrence frequencies of 3 × 3 bitmap patterns in the binary image. The distance between the binary images was measured based on the frequency histograms of the bitmap patterns. Five frequently-used distance measures were evaluated for their performance in determining the distance between histograms based on the phylogeny of 31 mitochondrial genome sequences. These include histogram intersection [10], Manhattan distance, Bhattacharyya distance [11], Jensen–Shannon divergence [12], and Kendall’s rank correlation coefficient [13].

## Methods

### Generating a binary image from a DNA sequence

We describe here the step-by-step procedure used to generate a binary image from a DNA sequence.

#### Graphical representation of a DNA sequence

**Figure 1:**
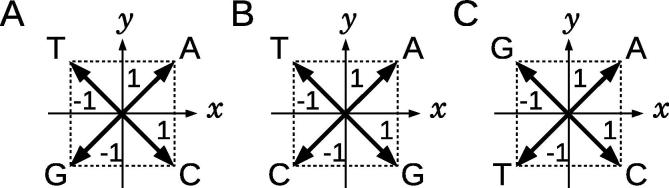
Three independent assignments of vectors on the *xy*-plane to individual nucleotides There are three independent assignments under the symmetries of 90-degree rotations and the inversion with respect to the vertical or horizontal axis. Four nucleotides A, T, G, and C are arranged counterclockwise on the *xy*-plane “ATGC” (**A**), “ATCG” (**B**), and “AGTC” (**C**). Assignment **A** is used throughout the study.

**Figure 2:**
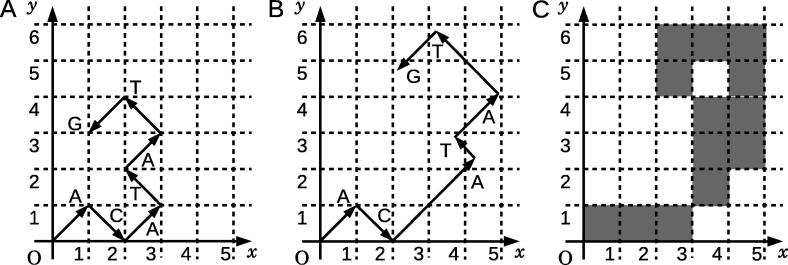
Generating a binary image of sequence “ACATATG” **A.** The primary graphical representation. **B.** The graphical representation modified with weighting factors. **C.** The generated binary image. Each grid represents an individual pixel of a binary image.

Firstly, we assigned 2D numerical vectors on the *xy*-plane, which are perpendicular or in opposite directions to each other, to A, T, G, and C. The number of independent variations of the assignments was 3!/2 = 3, including the assignments that can be transformed into each other by 90-degree rotations or the inversions with respect to vertical or horizontal axes (Figure 1). We chose the assignment presented in Figure 1A, where nucleotides A and T are placed in the upper quadrants, and G and C in the lower ones, so that the GC content of a DNA sequence can be grasped easily from the resultant graphical representation. Note that, the assignment presented in Figure 1B is also acceptable, but better results are obtained with the former assignment as shown in Figure 1A. Therefore, the assignment given in Figure 1A is adopted throughout this article. Then, a 2D graph can be drawn by consecutively connecting the vectors assigned to the nucleotides of a DNA sequence. A graphical representation of a sequence, “ACATATG”, is represented in Figure 2**A**.

#### Multiplying weighting factors

In order to extract potential information conveyed by individual nucleotides, we introduced weighting factors, based on a Markov chain model, into the process of binary images generation [14]. To emphasize rare patterns that appear in genome sequences, we used self-information *I*(*E*), the amount of information that is received when a certain event *E* occurs, as the weighting factor. Let *P*(*E*) be the probability that event *E* occurs, *I*(*E*) is defined as *I*(*E*) = −log_2_ *P*(*E*) in bit units. A trajectory for each genome sequence in a 2D plane is drawn as follows:

(1) $$R_{i}=\overset{i}{\underset{k=1}{\sum }}w_{k}V_{k}\text{,}$$

where $R_{i}$ is the coordinate of the *i*th point on the trajectory, $V_{k}$ is the vector assigned to the *k*th nucleotide of the genome sequence, and $w_{k}$ is the corresponding weighting factor *I*(*E*). Here, we defined *P*(*E*) according to the second order Markov chain. The probability that nucleotide *z* occurs after a pair of nucleotides *xy* (*x*, *y*, *z* ∈ {A, T, G, C}) is calculated using

(2) $$P(z|\mathrm{xy})=\frac{N_{\mathrm{xyz}}}{\sum_{s\in \{\text{A,T,G,C}\}}N_{\mathrm{xys}}}\text{,}$$

where *N_xyz_* and *N_xys_* (*z*, *s* ∈ {A, T, G, C}) are the numbers of occurrence of triplets *xyz* and *xys*, respectively, which were measured in all analyzed DNA sequences.

Procedure of the graphical representation with weighting factors is illustrated in Figure 2. Suppose that *P*(A|AC), *P*(T|CA), *P*(A|AT), *P*(T|TA), and *P*(G|AT) are 0.20, 0.66, 0.41, 0.31, and 0.44, respectively, the weighting factors are calculated as 2.3, 0.60, 1.3, 1.7, and 1.2, respectively. The first two in a series of vectors represented in Figure 2A are drawn without weighting factors, because the corresponding weighting factors are not available. Due to the occurrence of “AC” in the preceding doublet, the third vector “A” is multiplied by 2.3, *i.e.*, the weighting factor calculated for *P*(A|AC). Similarly, the remaining vectors are multiplied by the corresponding weighting factors. As a result, the graphical representation of sequence “ACATATG” is modified as shown in Figure 2**B**.

#### Generating a binary image

A binary image is a digitized image in which each pixel is set as either 0 or 1 typically plotted in *white* and *black*, respectively. In this study, we set “1” for the pixels that include at least a portion of a vector, and “0” for all other cases. The graphical representation of a DNA sequence shown in Figure 2B was then converted to a binary image (Figure 2**C**).

### Local patterns of pixels of a binary image

Local pattern is defined as a bitmap image composed of the adjacent pixels in a certain size of window. Each pixel of a binary image has two value options (0 and 1), and therefore, the number of local patterns is $2^{n}$, where *n* is the number of pixels in a window. Very large windows are dominated by white pixels, while the windows that are too small cannot have enough variations to express a DNA sequence. Therefore, for this study we chose the window size of 3 × 3, where the number of the local patterns is 2^9^ = 512. Local pattern with pixels that are all white was not included in the local pattern histograms, because it represents the empty background of the images.

**Figure 3:**
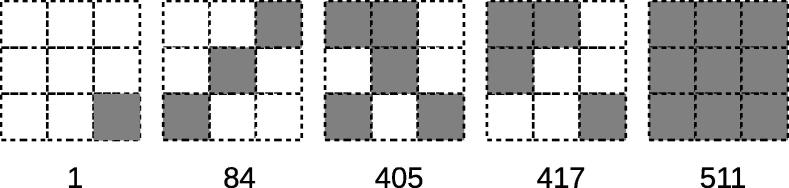
Five examples of local patterns with their serial numbers below Each grid represents an individual pixel of a binary image. The serial numbers are given by lining up the pixels from the upper left corner to the lower right and interpreting them as a binary number (“0” for white and “1” for black pixels, respectively), with the upper left corner being the highest bit.

A serial number was assigned to each local pattern by lining up the pixels from the upper left corner to the lower right with the upper left corner being the highest bit. Pixels were expressed as binary numbers (“0” for white and “1” for black). Figure 3 shows five examples of the local patterns of window size 3 × 3, with their serial numbers shown below, which will help readers to understand the relationships between the local patterns and their serial numbers.

### Counting the occurrence frequencies of the local patterns

Occurrence frequencies of the local patterns were counted by sliding a 3 × 3 window by one pixel per move over the binary image. If the trajectory of the graphical representation of a DNA sequence is like a *random walk*, the average distance between the origin and the terminus of the trajectory would be *O*(*L*^1/2^), where *L* is the sequence length, and the rectangle area covering the whole trajectory is proportional to *L*. Consequently, the computation time to count the occurrence frequencies would become *O*(*L*). However, this is not the case in reality. In most cases, the trajectory is not like a random walk but is a curved line of length *O*(*L*). Therefore, the computation time to count the occurrence frequencies becomes $O(L^{2})$, which is the equivalent of the pairwise sequence alignment.

**Figure 4:**
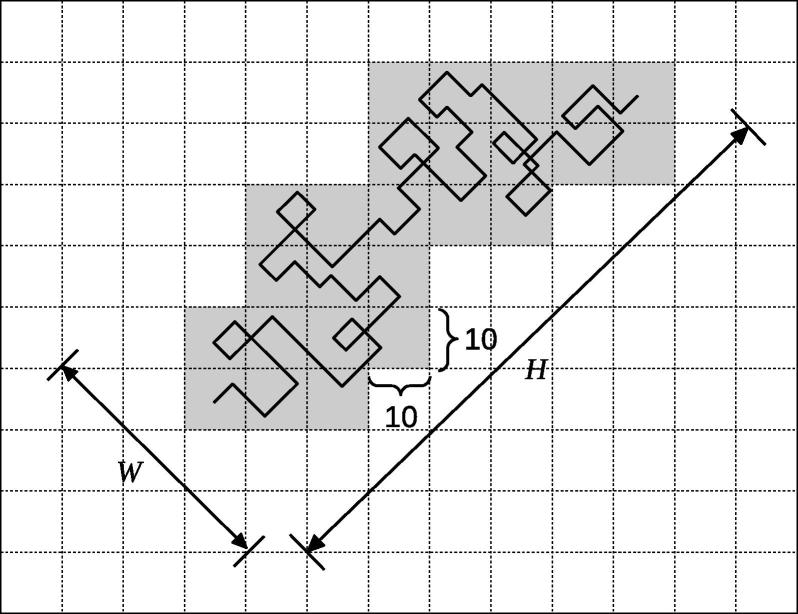
Counting areas on a binary image Counting the occurrence frequencies of local patterns is performed only on the gray-colored areas, which include at least one black pixel. *W* and *H* are the width and the length of the aggregation of the colored areas, respectively.

To reduce the computation time, we developed a new method for counting the occurrence frequencies. As shown in Figure 4, a binary image is divided into squares of 10 × 10 pixels and squares containing at least one black pixel are marked when generating the binary image (gray squares in Figure 4). Occurrence frequencies of the local patterns are counted only in the marked areas. Let *W* and *H* be the width and length of the aggregate region of the marked areas, respectively, then the computation time of counting is estimated approximately as *O*(*WH*). For the binary images generated from mitochondrial genomes, *W* is almost independent of the genome length *L*, and *H* is proportional to *L* (see Results and discussion). Therefore, the computation time is reduced to *O*(*L*). There remains room for improvement by reducing the numerical coefficient. For instance, this coefficient can be reduced using a variable grid size instead of the fixed size of 10 × 10, which can be adjusted adaptively for the individual counting areas. In addition, since the counting procedure is performed independently on the individual areas of the binary images, parallel processing can be implemented for the counting procedure, which would further boost the performance of our method.

### Distance measures between local pattern histograms

Different measures can be used to estimate the similarity/dissimilarity between two histograms. In this study, we chose five commonly-used measures and compared their performance in the distance measures between the local pattern histograms, which could be employed in our method. These include histogram intersection (HI) [10], Manhattan distance (MD), Bhattacharyya distance (BD) [11], Jensen–Shannon divergence (JS) [12], and Kendall’s rank correlation coefficient (*τ*) [13]. Their definitions and the modifications in this study are listed in Table 1. *p_i_* and $q_{i}$ are the occurrence frequencies of the local pattern of serial number *i* in histograms *P* and *Q*, respectively; and *N* is the largest serial number of the local patterns (*i.e.*, *N* = 511 for 3 × 3 pixel local patterns). Note that in the calculation of the distances, the occurrence frequencies are normalized to be $\sum_{i=1}^{N}p_{i}=\sum_{i=1}^{N}q_{i}=1$.

### Evaluation of the calculated distances among the local pattern histograms

To evaluate the calculated distances, we reconstructed phylogenetic trees from the distance matrix based on each distance measure listed in Table 1. We then computed the Robinson–Foulds (R–F) distances [16] between our trees and a reference tree reconstructed using ClustalW based on the multiple sequence alignment of the mitochondrial genome sequences. The R–F distances were calculated by TREEDIST program in Phylogeny Inference Package PHYLIP [17].

## Results and discussion

### Genome sequences analyzed

Mitochondrial genome sequences of 31 mammalian species (Table 2) with their respective accession numbers and lengths, were obtained from the NCBI GenBank. These species were selected for comparison according to Huang et al. [6], except ape, Sumatran orangutan, goat, and giant panda genomes, for simplicity. Mitochondrial genomes are widely used to study genome evolution and phylogenetic inference, which show a high mutation rate compared with the nuclear genomes and a nearly uniform size across mammalian species [18].

### Weighting factors calculated from the occurrence frequencies of trinucleotides

We counted the number of occurrences of every tri-nucleotide in all the mitochondrial genome sequences listed in Table 2 by sliding a window of length three one nucleotide per move, and calculated the weighting factors as described in the Methods section. The occurrence frequencies of all tri-nucleotides detected in each genome sequence are listed in Table S1. The obtained weighting factors are shown in Table 3. The high value of weighting factors indicate that the corresponding tri-nucleotides rarely occur in the genome sequences, whereas low value of weighting factors indicates that the corresponding tri-nucleotides occur frequently in the genome sequences. To validate the robustness of the weighting factors shown in Table 3, we randomly selected 15 species (out of the original 31 genome sequences) and recalculated the weighting factors. We repeated this trial 100 times and obtained the average values very similar to those presented in Table 3, with the maximum standard deviation of 0.07 (data not shown). These results suggest that the weighting factors shown in Table 3 represent a snapshot of a comprehensive picture of the mammalian mitochondrial genomes examined.

### Graphical representations of mitochondrial genomes

**Figure 5:**
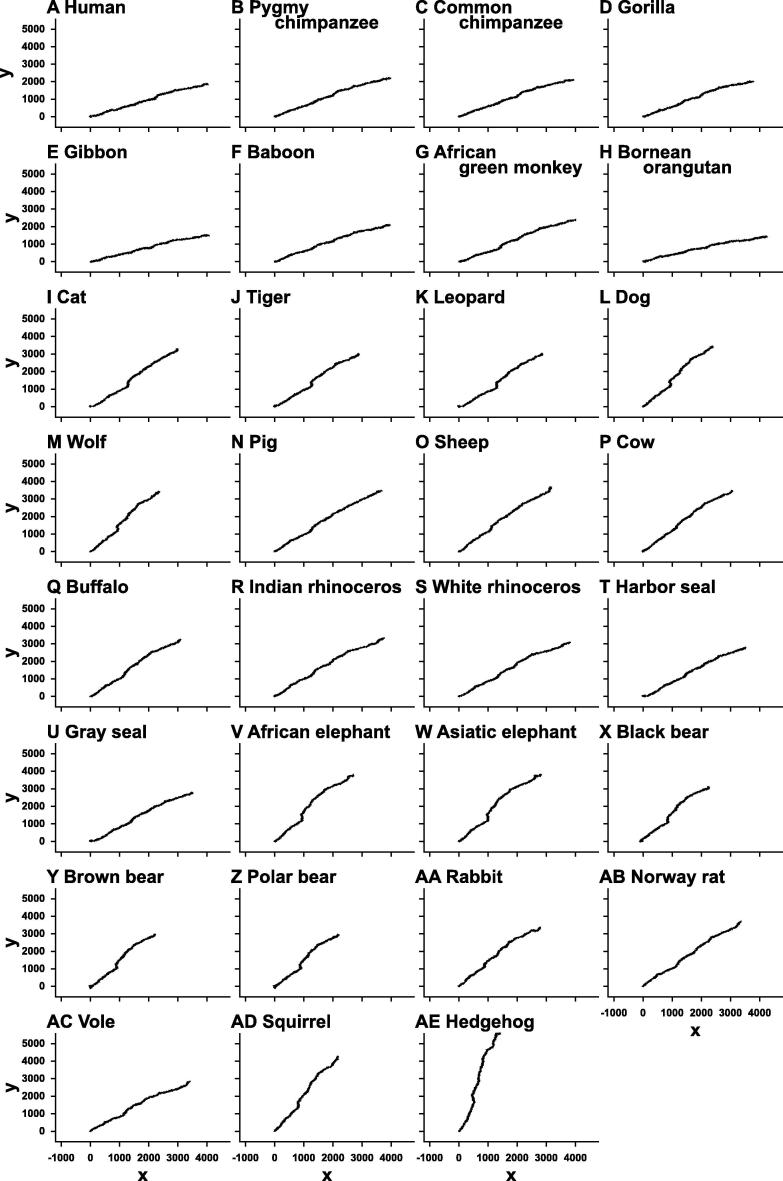
Graphical representation of mitochondrial genomes of 31 mammalian species without weighting The graphs are drawn on the *xy*-plane without weighting.

**Figure 6:**
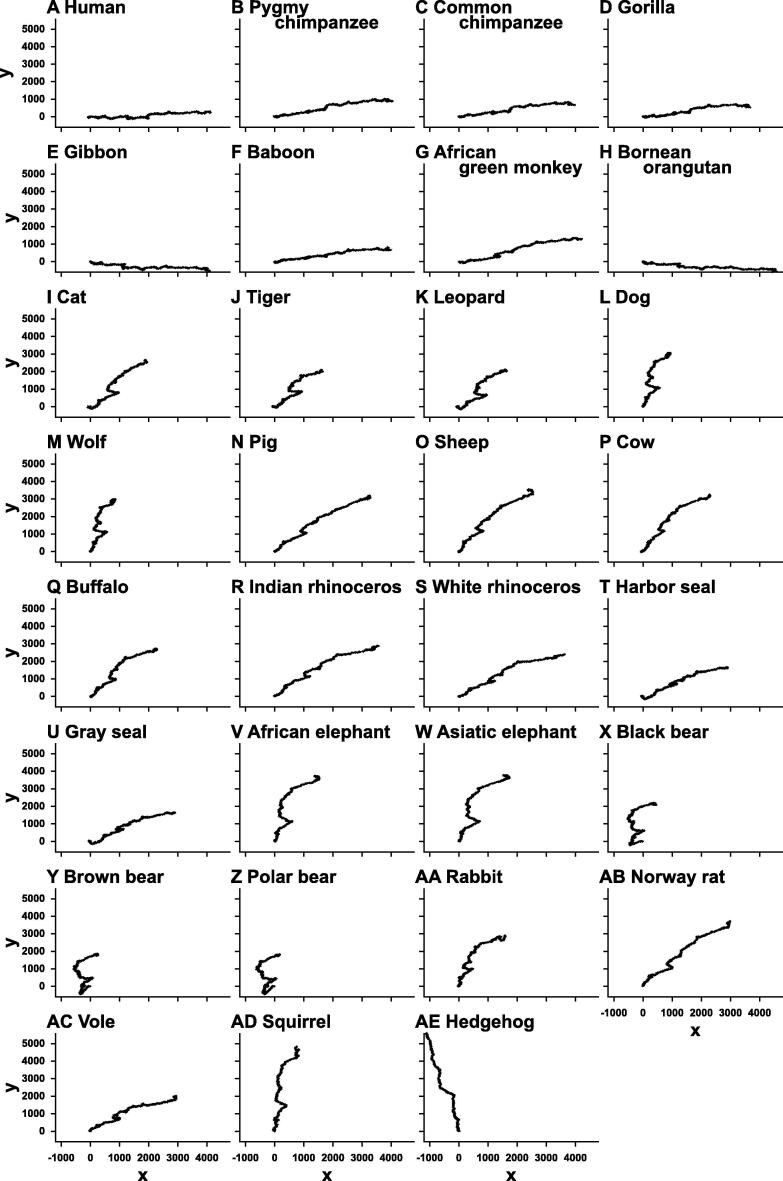
Graphical representation of mitochondrial genomes of 31 mammalian species with weighting The graphs are drawn on the *xy*-plane with weighting. The trajectories among closely-related species, such as primates, cats, elephants, bears, and so on, are similar to each other. On the other hand, trajectories between the species in different orders are distinct.

Graphical representations of the 31 mammalian mitochondrial genomes were drawn by our method without (Figure 5) and with weighting (Figure 6), respectively. The trajectories on the graphs in Figure 5 look very similar to each other, except for slight differences of their gradients. On the other hand, closely-related species, such as primates, cats, elephants, and bears, share similar trajectories, whereas trajectories between distant species are different (Figure 6). Comparing the graphs with and without weighting indicated that weighting makes it possible to distinguish even between the graphs of closely-related species. This demonstrates the effectiveness of our method of graphical representation for the visual inspection of the sequence similarities.

### Local pattern histograms counted on the graphical representations

We counted the occurrence frequencies of the local patterns for the 31 mammalian species, and constructed the local pattern histograms to explore which patterns are dominant. These histograms are shown in Figure S1. Local patterns 1, 4, 10, 34, 64, 84, 136, 160, 256, and 273 are detected more than 1000 times in each genome sequence. These local patterns are frequent because all vectors assigned to the nucleotides are on the diagonal lines of the *xy*-plane (Figure 1), and the binary images do not contain many black pixels. These frequent local patterns are depicted in Figure S2.

### Construction of phylogenetic trees based on the local pattern histograms

**Figure 7:**
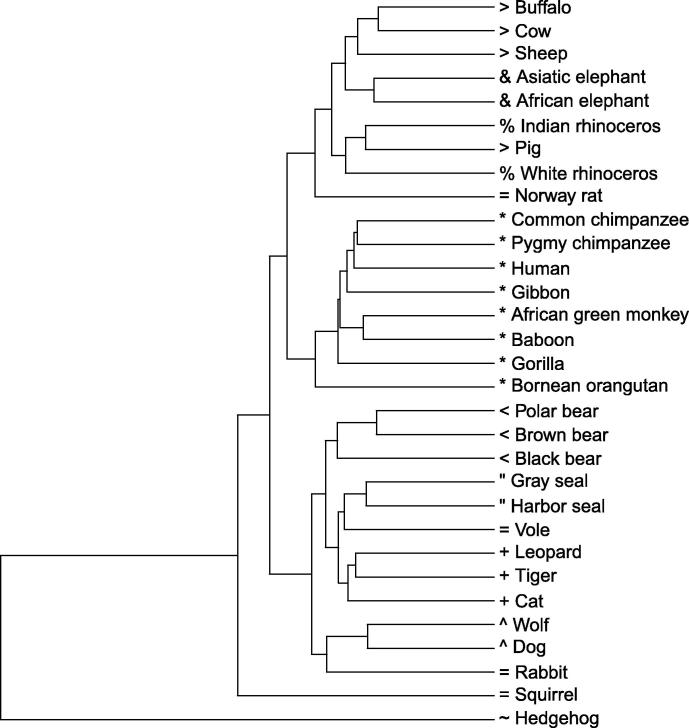
Phylogenetic tree reconstructed by our method based on HI and MD The tree is reconstructed using UPGMA algorithm based on the distance matrix calculated by HI and MD. Scale bars are not indicated, since absolute values of distances derived from the distance measures used in this study are less informative. HI, histogram intersection; MD, Manhattan distance. Tips before species’ names are indicated as follows: “^∗^”, primates; “=”, glires (rodents and rabbit); “+”, cats; “^”, dogs; “>”, cetartiodactyla (bovines and pig); “%”, rhinoceros; ““”, seals; “<”, bears; “&”, elephants; and “∼”, hedgehog.

A phylogenetic tree was reconstructed to evaluate our method from the calculated distance matrix based on HI, using Unweighted Pair Group Method with Arithmetic mean (UPGMA) (Figure 7). The same tree is obtained using MD. Phylogenetic trees generated based on BD, JS, and Kendall’s *τ* are presented in Figure S3. All the trees were drawn by the statistical analysis software R, using “ape” package. The tree based on HI (and MD) seems to be reconstructed well, since primates, elephants, cats, bears, *etc*. are located in their respective clades (Figure 7). On the other hand, some species are located in inadequate places on the trees based on the other distance measures. For instance, sheep is separated from buffalo–cow pair in the tree built using BD as shown in Figure S3A, pig and white rhinoceros are included in primates, and leopard is separated from cat–tiger pair in the tree built using Kendall’s *τ* as shown in Figure S3B.

Table 4 shows Pearson’s correlation coefficients calculated between the distance matrices based on the five distance measures. HI–MD pair and BD–JS pair are strongly correlated, confirming the topological similarity of the resultant phylogenetic trees in Figures 7 and S3. To quantitatively evaluate the phylogenetic trees built in this study, we measured the Robinson–Foulds (R–F) distances [16] between the trees we constructed and a reference tree reconstructed using ClustalW based on the multiple sequence alignment of the mitochondrial genome sequences (Figure S4). As shown in Table 5, among the five distance measures, trees built with HI and MD had the lowest R–F distances with that built with ClustalW, suggesting their superior performance in building phylogenetic trees.

We compared our tree reconstructed by HI (and MD) in Figure 7 with those given by Huang et al. [6] and Yu et al. [19] for further evaluation. The overall configuration of the tree we constructed roughly agrees with the trees shown in these studies, except for the position of hedgehog. Krettek et al. [20] performed a phylogenetic analysis using concatenated sequences of 13 mitochondrial protein-coding genes of nine mammals, including human, harbor seal, cow, and hedgehog, and identified the position of hedgehog as basal relative to the other species included. Our results are consistent with those obtained by Krettek et al. [20], rather than Huang et al. [6] and Yu et al. [19].

## Consideration of the execution time

We further compared the execution time for handling 31 mammalian mitochondrial genomes, between ClustalW and our method. The most time-consuming process in ClustalW is multiple sequence alignment, whereas for our method, counting the occurrence frequencies of the local patterns in the binary images is the most time-consuming step. It takes around 60 min to perform the multiple sequence alignment in ClustalW, using Intel Core i5-4690 CPU of 3.50 GHz. Nonetheless, with the same PC configuration, counting the occurrence frequencies takes about 260 s in our method, representing approximately a 14-fold decrease in execution time. Other steps in our method, such as calculating weighting factors, making distance matrices, and drawing phylogenetic trees, require several seconds at most for each.

Execution time depends on the number of sequences involved. Multiple sequence alignment by ClustalW is performed by combining pairwise sequence alignment of all the pairs of the sequences involved. Therefore, the dependency of the execution time on the number of sequences *S* is $O(S^{2})$ for ClustalW. For the method we present in this paper, this dependency is *O*(*S*), since the counting of the occurrence frequencies of local patterns in our method is independently performed on the sequences involved.

## Conclusion

In this study, we proposed a novel alignment-free method for the estimation of similarities between DNA sequences. In this method, we express DNA sequences as binary images by replacing individual nucleotides with 2D vectors and connecting them successively. We counted the occurrence frequencies of 3 × 3 bitmap patterns in the binary images, and measured the distances between them based on the frequency histograms of the bitmap patterns. We chose five frequently-used distance measures to estimate similarity/dissimilarity between two histograms: histogram intersection, Manhattan distance, Bhattacharyya distance, Jensen–Shannon divergence, and Kendall’s rank correlation coefficient.

We compared our phylogenetic trees with a reference tree reconstructed by ClustalW for mitochondrial genomes of 31 mammalian species. Among the five distance measures, histogram intersection and Manhattan distance showed the best performance in terms of Robinson–Foulds distance between the phylogenetic trees. In addition, a more appropriate positioning of hedgehog in the phylogenetic tree was obtained when compared with the phylogenetic tree reconstructed by Huang et al. [6] and Yu et al. [19].

The most time-consuming step in our method is counting the occurrence frequencies of local patterns. Its time complexity is *O*(*L*) for sequence length *L*, which is practical even for very long sequences.

## Authors’ contributions

YK carried out the data analysis and drafted the manuscript. SM conceived the project, participated in study design and coordination, and revised the manuscript. All authors read and approved the final manuscript.

## Competing interests

The authors have declared that no competing interest exists.

## Figures and Tables

**Table 1:** Five distance measures used in this study

**Distance measure**	**Definition**	**Range**	**Note**
HI	$\text{HI}(P\text{,}Q)=\sum_{i=1}^{N}\min(p_{i}\text{,}q_{i})$	0–1	HI is converted to $D_{\text{HI}}(P\text{,}Q)=1-\text{HI}(P\text{,}Q)$ for calculating distances; HI = 1 when *P* is identical to *Q*
MD	$D_{\text{MD}}(P\text{,}Q)=\sum_{i=1}^{N}|p_{i}-q_{i}|$	0–2	*D*_MD_ is also known as City block distance or L_1_-norm *D*_MD_ = 0 when *P* is identical to *Q*
BD	$\text{DIV}(P\text{,}Q)=\sum_{i=1}^{N}\sqrt{p_{i}q_{i}}$	0–1	DIV is converted to *D*_BD_(*P*, *Q*) = −ln DIV (*P*, *Q*) for calculating distances DIV = 1 when *P* is identical to *Q*
JS	$D_{\text{JS}}(P\text{,}Q)=\frac{1}{2}\sum_{i=1}^{N}p_{i}\log_{2}\;\frac{2p_{i}}{p_{i}+q_{i}}+\frac{1}{2}\sum_{i=1}^{N}q_{i}\log_{2}\;\frac{2q_{i}}{p_{i}+q_{i}}$	0–1	*D*_JS_ is a symmetrized and smoothed version of Kullback–Leibler divergence [15]. Local patterns with $p_{i}=q_{i}=0$ are excluded from the calculation. *D*_JS_ = 0 when *P* is identical to *Q*
Kendall’s *τ*	$\tau =\frac{X-Y}{\sqrt{X+Y+r}\sqrt{X+Y+s}}$	−1–1	*τ* is converted to $D_{\tau }(P\text{,}Q)=1-\frac{\tau +1}{2}$ for calculating distances The corresponding *i*,*j* pairs are excluded from the computation when $p_{i}=p_{j}$ and $q_{i}=q_{j}$ *τ* = 1 when the rank orders of $p_{i}\text{s}$ and $q_{i}\text{s}$ are completely in agreement with each other

*Note: p_i_* and $q_{i}$ are the occurrence frequencies of the local pattern of serial number *i* in histograms *P* and *Q*, respectively. *X* is the number of the concordant *i*,*j*^0^(*i* > *j*) pairs in which $(p_{i}-p_{j})(q_{i}-q_{j})>0$ is satisfied; *Y* is the number of the discordant pairs in which $(p_{i}-p_{j})(q_{i}-q_{j})<0$ is satisfied; *r* is the number of the tie pairs in which $p_{i}=p_{j}$ and $q_{i}\;\neq \;q_{j}$ are satisfied; and *s* is the number of the other type of tie pairs in which $p_{i}\;\neq \;p_{j}$ and $q_{i}=q_{j}$ are satisfied. HI, histogram intersection; MD, Manhattan distance; BD, Bhattacharyya distance; JS, Jensen–Shannon divergence; Kendall’s *τ*, Kendall’s rank correlation coefficient; DIV, divergence.

**Table 2:** Mitochondrial genomes analyzed

**GenBank accession No.**	**Species**	**Length (bp)**
V00662	Human	16,569
D38116	Pygmy chimpanzee	16,563
D38113	Common chimpanzee	16,554
D38114	Gorilla	16,364
X99256	Gibbon	16,472
Y18001	Baboon	16,521
AY863426	African green monkey	16,389
D38115	Bornean orangutan	16,389
U20753	Cat	17,009
EF551003	Tiger	16,990
EF551002	Leopard	16,964
U96639	Dog	16,727
EU442884	Wolf	16,774
AJ002189	Pig	16,680
AF010406	Sheep	16,616
V00654	Cow	16,338
AY488491	Buffalo	16,355
X97336	Indian rhinoceros	16,829
Y07726	White rhinoceros	16,832
X63726	Harbor seal	16,826
X72004	Gray seal	16,797
AJ224821	African elephant	16,866
DQ316068	Asiatic elephant	16,902
DQ402478	Black bear	16,868
AF303110	Brown bear	17,020
AF303111	Polar bear	17,017
AJ001588	Rabbit	17,245
X88898	Hedgehog	17,447
X14848	Norway rat	16,300
AF348082	Vole	16,312
AJ238588	Squirrel	16,507

**Table 3:** Calculated weighting factors for each tri-nucleotide

**1^st^ base**	**2^nd^ base**				**3^rd^ base**
**A**	**C**	**G**	**T**
A	1.64	1.66	2.08	1.68	A
1.90	1.80	1.58	1.94	C
2.94	3.28	2.22	2.85	G
1.84	1.76	2.23	1.79	T

C	1.68	1.80	1.82	1.37	A
1.91	1.70	1.83	2.03	C
2.88	3.57	2.43	3.01	G
1.80	1.65	2.00	2.03	T

G	1.57	1.70	1.57	1.33	A
2.07	1.51	1.87	2.23	C
2.30	3.95	2.45	2.73	G
2.17	1.86	2.27	2.07	T

T	1.71	1.54	1.46	1.55	A
2.01	1.82	2.12	1.87	C
2.56	3.38	2.31	3.02	G
1.85	1.85	2.29	1.94	T

*Note:* Weighting factors are calculated with the 31 mammalian mitochondrial genomes.

**Table 4:** Pearson’s correlation coefficients of the five distance measures

	**HI**	**MD**	**BD**	**JS**
MD	0.99933			
BD	0.97994	0.97277		
JS	0.98091	0.97389	0.99997	
*τ*	0.94098	0.93999	0.93992	0.9405

*Note:* HI, histogram intersection; MD, Manhattan distance; BD, Bhattacharyya distance; JS, Jensen–Shannon divergence; *τ*, Kendall’s *τ*.

**Table 5:** Robinson–Foulds distances calculated for the five distance measures

**Distance measure**	**Robinson–Foulds distance**
Histogram intersection	30
Manhattan distance	30
Bhattacharyya distance	34
Jensen–Shannon divergence	34
Kendall’s *τ*	46

*Note:* Robinson–Foulds distances are calculated between reference tree built using ClustalW and the trees built based on the five distance measures in this study.

## Supplementary material

Supplementary Figure S1 Local pattern histograms of mitochondrial genomes of 31 mammalian species. Raw occurrence frequencies are plotted.

Supplementary Figure S2 Frequently-occurring local patterns. The local patterns that are detected more than 1000 times in every genome sequence are listed. Each grid represents an individual pixel of a binary image. The serial numbers are given by lining up the pixels from the upper left corner to the lower right and interpreting them as a binary number (“0” for white and “1” for black pixels, respectively), with the upper left corner being the highest bit.

Supplementary Figure S3 Phylogenetic trees reconstructed by our method based on BD, JS, and Kendall’s *τ*. These trees are reconstructed using UPGMA algorithm based on distance matrices calculated by Bhattacharyya distance and Jensen–Shannon divergence (A) and Kendall’s *τ* (B). BD, Bhattacharyya distance; JS, Jensen–Shannon divergence; Kendall’s τ, Kendall’s rank correlation coefficient. Tips before species’ names are indicated as follows: “^∗^”, primates; “=”, glires (rodents and rabbit); “+”, cats; “^”, dogs; “>”, cetartiodactyla (bovines and pig); “%”, rhinoceros; ““”, seals; “<”, bears; “&”, elephants; and “∼”, hedgehog.

Supplementary Figure S4 Reference tree reconstructed by ClustalW. The tree is reconstructed based on multiple sequence alignment of the 31 mitochondrial mammalian genome sequences. Tips before species’ names are indicated as follows: “^∗^”, primates; “=”, glires (rodents and rabbit); “+”, cats; “^”, dogs; “>”, cetartiodactyla (bovines and pig); “%”, rhinoceros; ““”, seals; “<”, bears; “&”, elephants; and “∼”, hedgehog.

Supplementary Table S1 The occurrence frequencies of all the tri-nucleotides detected in each species.

## Footnotes

Supplementary material associated with this article can be found, in the online version, at http://dx.doi.org/10.1016/j.gpb.2015.09.007.
