# Supplementary figures and images for: Similarity Estimation Between DNA Sequences Based on Local Pattern Histograms of Binary Images
Source: Genomics Proteomics Bioinformatics. 2016 Apr 27;14(2):103–12. doi: 10.1016/j.gpb.2015.09.007 (PMC4880953; doi:10.1016/j.gpb.2015.09.007)

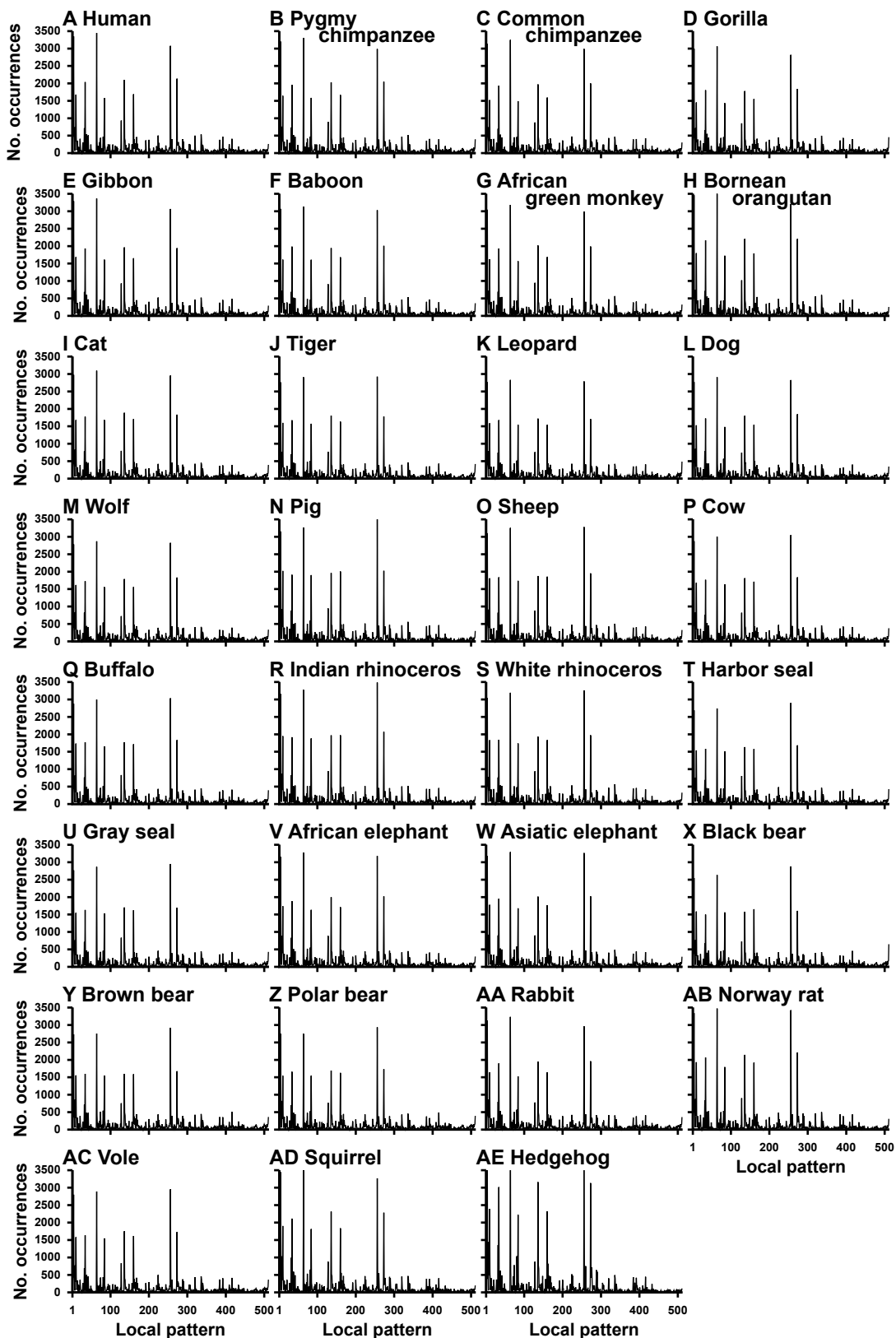

Supplement: Supplementary Figure S1 — Local pattern histograms of mitochondrial genomes of 31 mammalian species. Raw occurrence frequencies are plotted. [file mmc1.pdf]

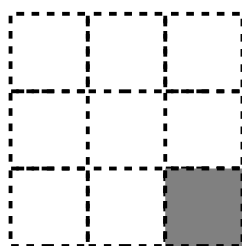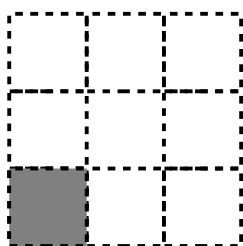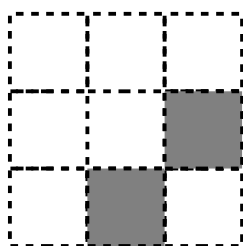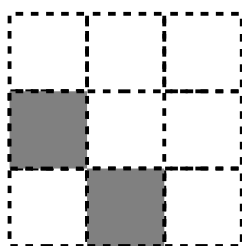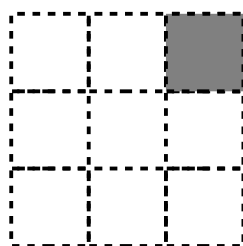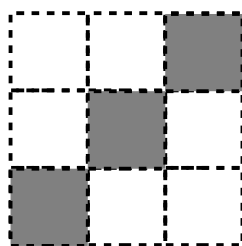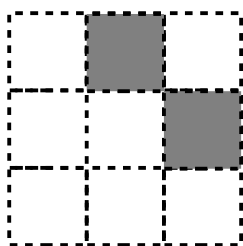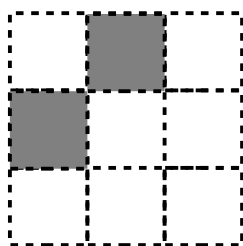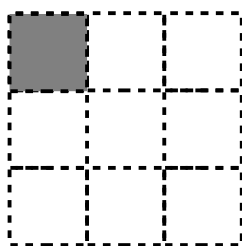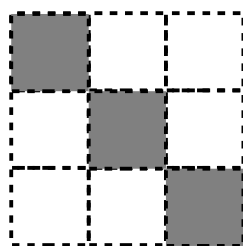

Supplement: Supplementary Figure S2 — Frequently-occurring local patterns. The local patterns that are detected more than 1000 times in every genome sequence are listed. Each grid represents an individual pixel of a binary image. The serial numbers are given by lining up the pixels from the upper left corner to the lower right and interpreting them as a binary number (“0” for white and “1” for black pixels, respectively), with the upper left corner being the highest bit. [file mmc2.pdf]

A

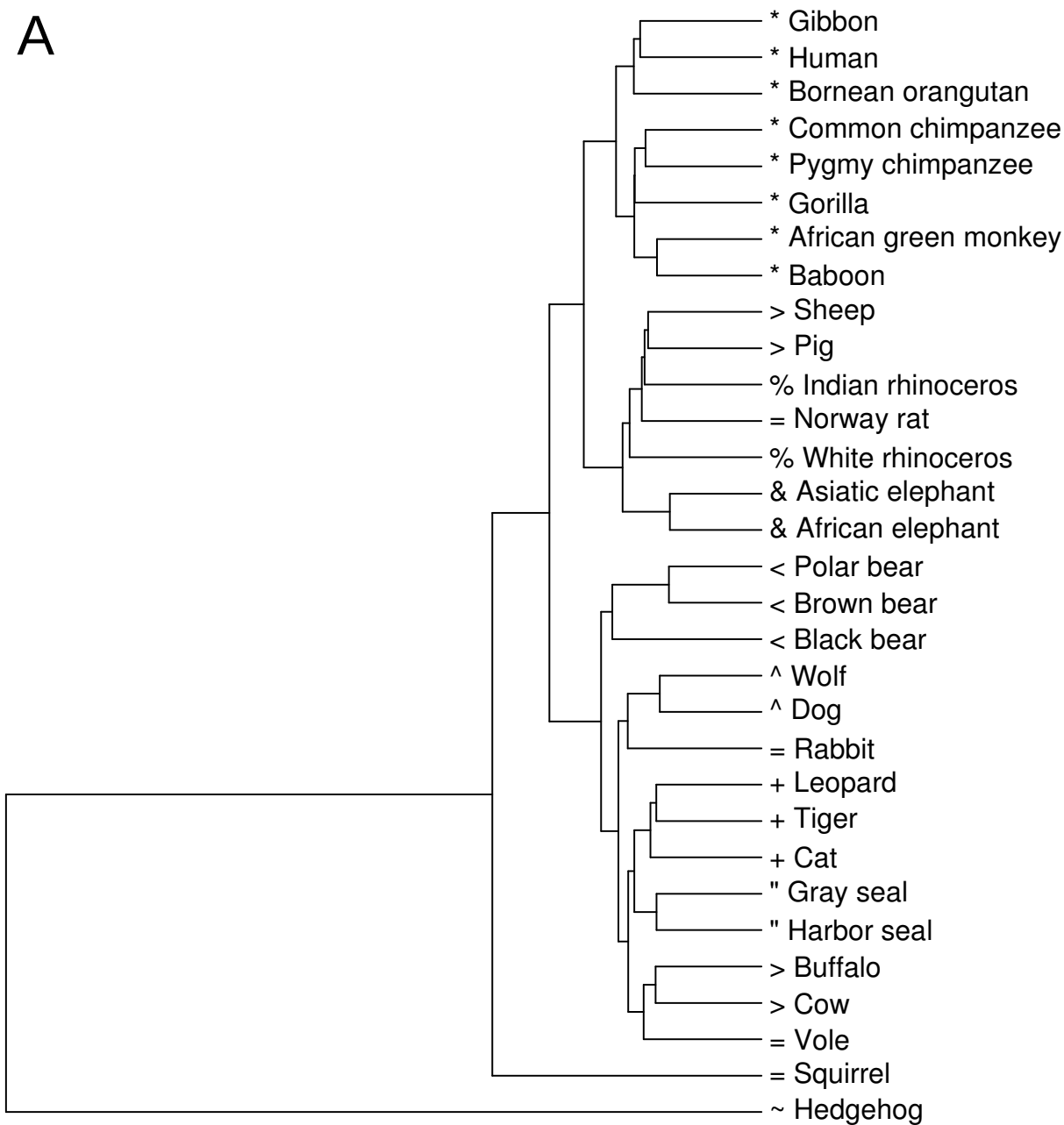

B

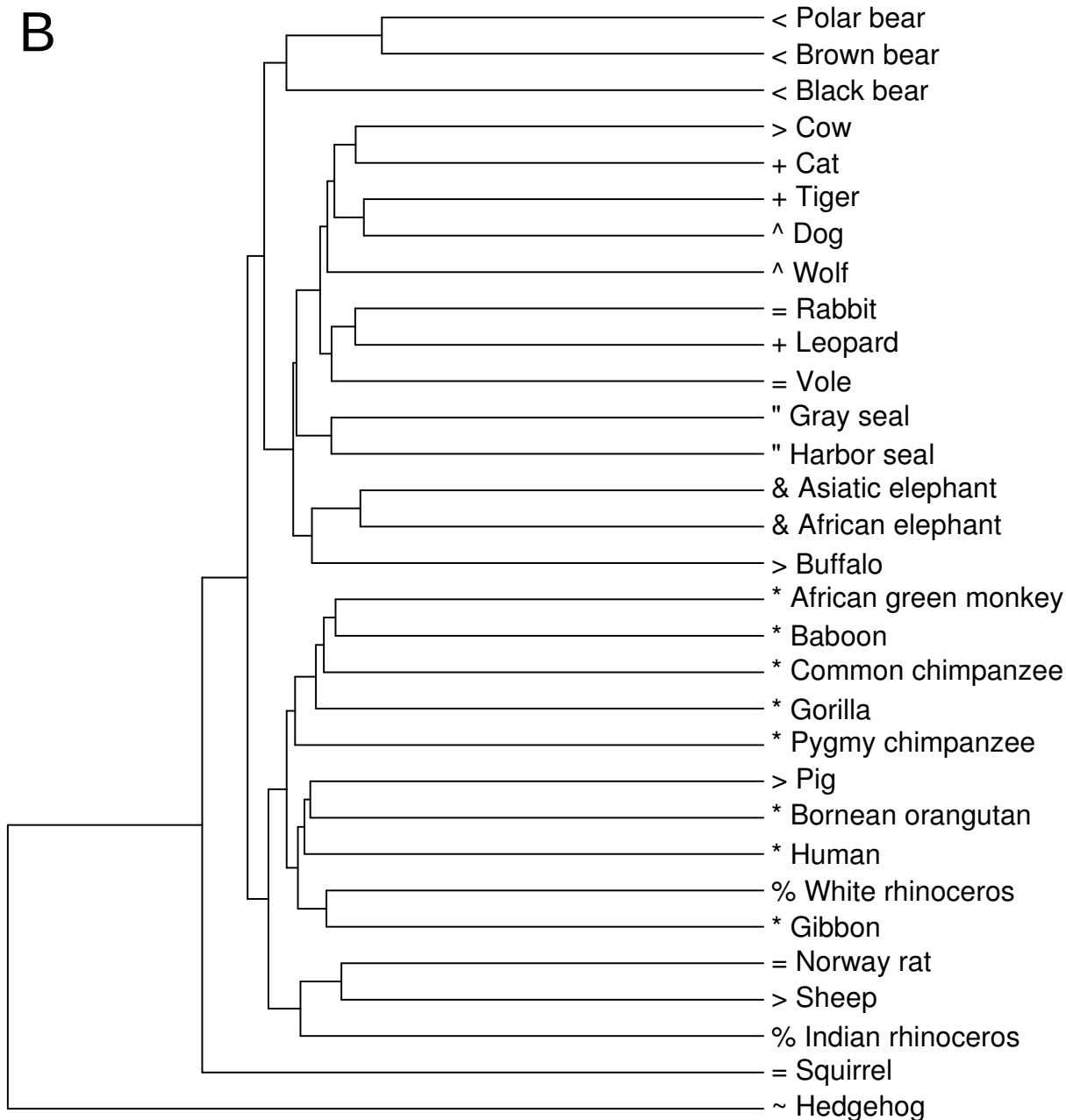

Supplement: Supplementary Figure S3 — Phylogenetic trees reconstructed by our method based on BD, JS, and Kendall’s τ. These trees are reconstructed using UPGMA algorithm based on distance matrices calculated by Bhattacharyya distance and Jensen–Shannon divergence (A) and Kendall’s τ (B). BD, Bhattacharyya distance; JS, Jensen–Shannon divergence; Kendall’s τ, Kendall’s rank correlation coefficient. Tips before species’ names are indicated as follows: “∗”, primates; “=”, glires (rodents and rabbit); “+”, cats; “^”, dogs; “>”, cetartiodactyla (bovines and pig); “%”, rhinoceros; ““”, seals; “<”, bears; “&”, elephants; and “∼”, hedgehog. [file mmc3.pdf]

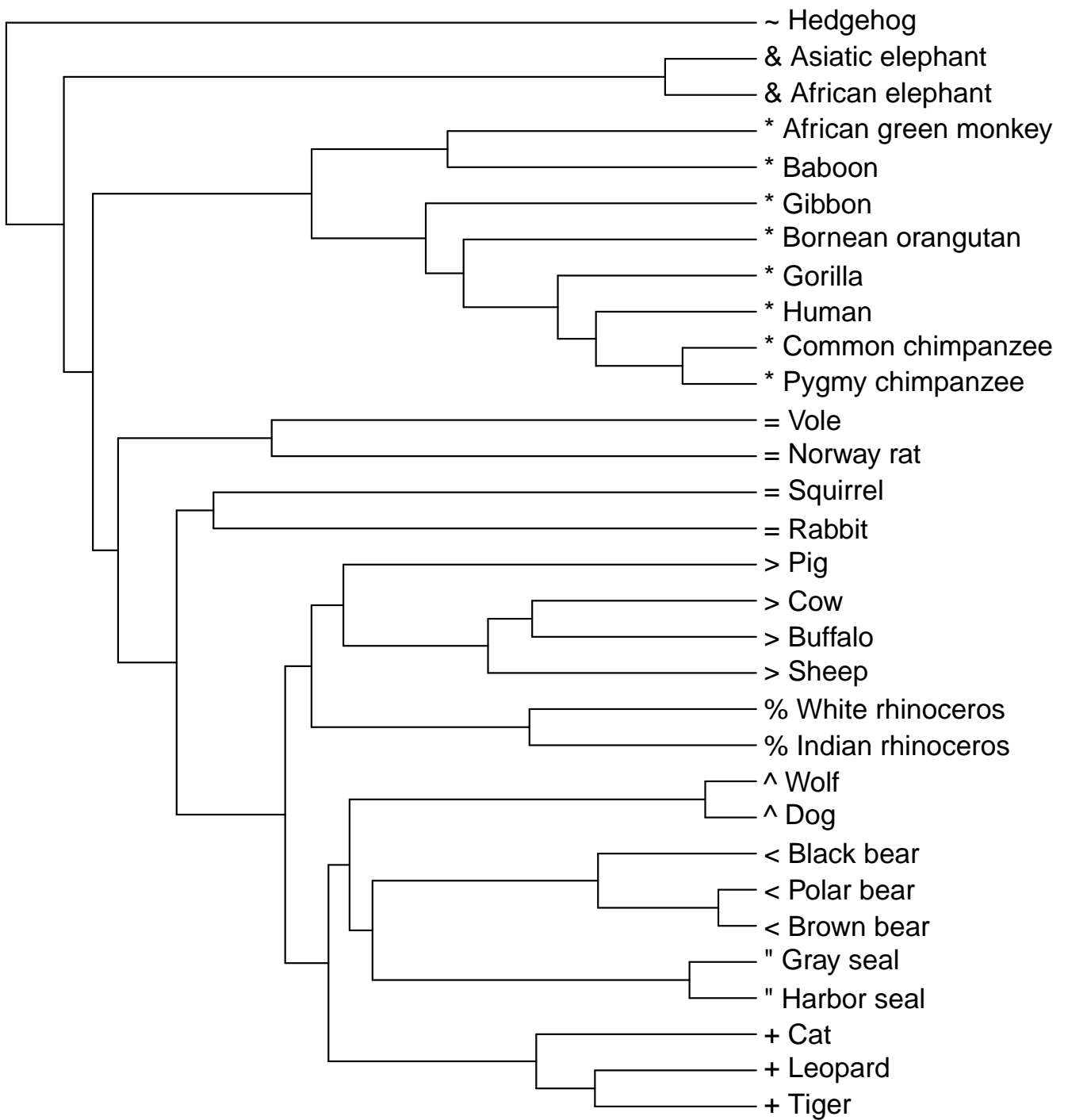

Supplement: Supplementary Figure S4 — Reference tree reconstructed by ClustalW. The tree is reconstructed based on multiple sequence alignment of the 31 mitochondrial mammalian genome sequences. Tips before species’ names are indicated as follows: “∗”, primates; “=”, glires (rodents and rabbit); “+”, cats; “^”, dogs; “>”, cetartiodactyla (bovines and pig); “%”, rhinoceros; ““”, seals; “<”, bears; “&”, elephants; and “∼”, hedgehog. [file mmc4.pdf]
